# Gastroparesis induced by glucagon-like peptide-1 receptor agonists: A systematic review of clinical features, diagnosis, management, and outcomes

**DOI:** 10.1371/journal.pone.0354497

**Published:** 2026-08-13

**Authors:** Tope Olubodun, Morenike Adedoyin Osundina, David Olubukunmi Soyoye, Natalia Maria Bim de Oliveira, Ayodeji Bamidele Olubodun, Oluwatoyin Olanrewaju Ogundele

**Affiliations:** 1 Department of Community Medicine and Primary Care, Federal Medical Center Abeokuta, Abeokuta, Ogun State, Nigeria; 2 Department of Medicine, University of Ibadan, Ibadan, Oyo State, Nigeria; 3 Department of Medicine, Obafemi Awolowo University, Ile-Ife, Osun State, Nigeria; 4 Ferring Pharmaceuticals, São Paulo, São Paulo, Brazil; 5 Accident and Emergency Department, Olabisi Onabanjo University Teaching Hospital, Sagamu, Ogun State, Nigeria; 6 Nats’oojeh Hospital and Health Centre, Fort St James, British Columbia, Canada; Charite Universitatsmedizin Berlin, GERMANY

## Abstract

**Background:**

Glucagon-like peptide-1 receptor agonists (GLP-1RAs) are widely used for the treatment of type 2 diabetes mellitus and obesity. Although gastrointestinal adverse effects are common, gastroparesis is an under-recognised but clinically important complication. This systematic review summarises published evidence on the presentation, diagnosis, management, and outcomes of gastroparesis associated with GLP-1RA therapy in real-world clinical practice.

**Methods:**

A systematic review was conducted in accordance with PRISMA 2020 guidelines. PubMed, Embase, Scopus, and Web of Science were searched from inception to October 2025 for reports of symptomatic gastroparesis associated with GLP-1RA use. Eligible studies included case reports, case series, observational studies, and clinical trials involving adults treated with GLP-1RAs for diabetes or weight loss. Data extraction focused on patient characteristics, GLP-1RA exposure, clinical presentation, diagnostic findings, management strategies, and outcomes. Methodological quality was assessed using the Joanna Briggs Institute checklist for case reports.

**Results:**

Twelve case reports describing 13 patients met inclusion criteria. Most patients were female and had type 2 diabetes mellitus, though cases also occurred in non-diabetic individuals. Semaglutide was the most frequently implicated agent, followed by liraglutide, dulaglutide, and exenatide. Symptom onset ranged from hours to several months after treatment initiation, commonly following dose escalation or inappropriate re-initiation. Presentations varied from acute to chronic. Reported symptoms included nausea, vomiting, abdominal pain, bloating, early satiety, and oral intolerance. Diagnostic evaluation often demonstrated gastric distension or retained gastric contents on imaging or endoscopy in the absence of mechanical obstruction. Gastric emptying scintigraphy confirms delayed emptying in selected cases. Management primarily involved supportive and symptomatic care. Discontinuation of the offending GLP-1RA led to symptom resolution in all reported patients.

**Conclusion:**

GLP-1 receptor agonists may induce or worsen gastroparesis in both diabetic and non-diabetic patients, particularly following rapid dose escalation or inappropriate dosing. Awareness of this association is essential for timely diagnosis and management. Withdrawal of the causative agent is central to treatment and is associated with favourable outcomes and reversibility of gastric dysfunction. Careful patient selection, gradual dose titration, and monitoring for persistent gastrointestinal symptoms are recommended to optimise the safe use of GLP-1RAs.

## Introduction

Glucagon-like peptide-1 receptor agonists (GLP-1RAs) are synthetic analogues of the endogenous incretin hormone glucagon-like peptide-1 (GLP-1), which plays a central role in regulating postprandial glucose excursions, enhancing satiety, and slowing gastric emptying [1]. Since the approval of the first GLP-1RA, exenatide, nearly two decades ago, several additional agents—including dulaglutide, liraglutide, lixisenatide, and semaglutide—have been developed and approved for clinical use [2]. GLP-1RAs have since become integral to the management of type 2 diabetes and obesity, with increasing evidence supporting their role as adjunctive therapies in cardiovascular disease.

The glucagon-like peptide-1 receptor (GLP-1R) is widely expressed across multiple tissues and organ systems, including the gastrointestinal tract, central nervous system, cardiovascular system, lungs, kidneys, skin, and vagus nerve, reflecting the pleiotropic effects of GLP-1 signalling [3,4]. In the cardiovascular system, GLP-1R activation is associated with reduced atherosclerotic progression, improvements in lipid profiles, lowering of blood pressure, enhancement of vascular endothelial function, and reductions in major adverse cardiovascular events [3]. In the central nervous system, GLP-1R activity has been shown to reduce neuroinflammation, enhance synaptic transmission, and support neuroprotection and neural regeneration [3].

Consistent with these widespread receptor-mediated effects, GLP-1RAs may be classified according to their pharmacokinetic properties and chemical structure. Based on duration of action, they are categorised as short-acting or long-acting agents [5]. Irrespective of duration, GLP-1RAs delay gastric emptying, improve glycaemic control through glucose-dependent stimulation of insulin secretion and suppression of glucagon release, and regulate appetite by promoting satiety. Short-acting agents include exenatide and lixisenatide, while long-acting agents include dulaglutide, exenatide long-acting release, liraglutide, and semaglutide [5,6]. GLP-1RAs may also be classified by chemical structure into GLP-1–based derivatives and exendin-4–based derivatives [5].

Diabetes mellitus and overweight/obesity are major global public health challenges that require effective management to reduce the risk of complications such as cardiovascular disease, chronic kidney disease, neuropathy, retinopathy, and diminished quality of life. Newer GLP-1RAs have demonstrated substantial efficacy in improving glycaemic control and achieving clinically meaningful weight reduction. However, their use is commonly associated with gastrointestinal adverse effects, including nausea, vomiting, and diarrhoea, which are typically dose-dependent and often diminish with continued therapy [3]. Gastroparesis—defined as delayed gastric emptying in the absence of mechanical obstruction [7]—is also a recognised adverse effect of GLP-1RA therapy and can significantly impair quality of life, with potential complications such as poor glycaemic control, malnutrition, dehydration, medication intolerance, and increased healthcare utilisation [8].

As the use of GLP-1RAs expands, gastrointestinal symptoms are frequently anticipated and regarded as transient, which may contribute to under-recognition of more severe manifestations such as gastroparesis. This is particularly relevant among individuals with long-standing diabetes, in whom the clinical features of GLP-1RA-associated gastroparesis may overlap with those of diabetic gastroparesis, leading to diagnostic uncertainty and delays in appropriate management.

Existing systematic reviews and meta-analyses have largely focused on quantifying the odds ratios and risk ratios of gastrointestinal adverse effects associated with GLP-1RA therapy [9–14]. Such approaches do not adequately capture gastroparesis induced by GLP-1RAs as a distinct clinical entity encountered in routine practice. Although reports of GLP-1RA-associated gastroparesis are increasingly described, the available evidence remains fragmented and has not been systematically synthesised to characterise clinical presentation, diagnostic approaches, management strategies, and outcomes. Without such synthesis, clinicians lack consolidated evidence to support recognition of this adverse effect and to distinguish it from more benign gastrointestinal intolerance. This systematic review is therefore justified to comprehensively synthesise published clinical evidence on gastroparesis associated with GLP-1RA therapy.

By collating data on patient characteristics, symptom patterns, diagnostic methods, management approaches, and outcomes, this review aims to clarify the clinical spectrum of this condition and identify recurring features that may inform safer prescribing, timely recognition, and appropriate clinical decision-making. As GLP-1RA use continues to expand globally, including for indications beyond diabetes, a clearer understanding of this uncommon but potentially significant adverse effect is essential to balance therapeutic benefits against gastrointestinal harm and to guide future research.

Accordingly, this systematic review synthesised published evidence on gastroparesis associated with glucagon-like peptide-1 receptor agonist therapy, addressing the following question: among adults treated with GLP-1RA for diabetes or obesity, what clinical features, diagnostic approaches, management strategies, and outcomes of gastroparesis have been reported in association with these agents?

## Methods

### Study design

This systematic review was conducted following the PRISMA 2020 guidelines [15] to identify clinical trials and observational studies reporting gastroparesis or delayed gastric emptying related to GLP-1RA use.

### Databases and search strategy

Electronic searches were conducted in PubMed, Embase, Scopus, and Web of Science from inception to 14^th^ October 2025. Each database was searched using a combination of controlled vocabulary (e.g., MeSH terms) and free-text keywords related to glucagon-like peptide-1 receptor agonists and gastroparesis. Boolean operators (“AND,” “OR”) were used to systematically combine synonyms for GLP-1 receptor agonists – including individual drug names – with terms describing gastroparesis, delayed gastric emptying, or impaired gastric motility. Search strategies were tailored to the indexing structure of each database to ensure comprehensive coverage.

The following combination of MeSH terms and free-text keywords were used:

**SEARCH STRATEGIES FOR DATABASES**

**PUBMED**

(“Glucagon-Like Peptide 1 Receptor Agonists”[Mesh] OR “GLP-1 receptor agonist*”[tiab] OR “GLP1 receptor agonist*”[tiab] OR “incretin mimetic*”[tiab] OR “liraglutide”[tiab] OR “semaglutide”[tiab] OR “dulaglutide”[tiab] OR “exenatide”[tiab] OR “lixisenatide”[tiab] OR “albiglutide”[tiab] OR “taspoglutide”[tiab] OR “efpeglenatide”[tiab] OR “benaglutide”[tiab]) AND (“Gastroparesis”[Mesh] OR “Gastric Emptying”[Mesh] OR gastroparesis[tiab] OR “delayed gastric emptying”[tiab] OR “gastric stasis”[tiab] OR “impaired gastric motility”[tiab] OR ileus[tiab])

Filters applied: Case Reports, Clinical Study, Clinical Trial, Observational Study.

**SCOPUS**

TITLE-ABS-KEY((“glucagon like peptide 1” OR “GLP-1 receptor agonist*” OR “incretin mimetic*” OR liraglutide OR semaglutide OR dulaglutide OR exenatide OR lixisenatide)

AND (gastroparesis OR “delayed gastric emptying” OR “gastric stasis”

OR “gastric emptying” OR “gastric motility” OR “gastric emptying time”))

**EMBASE**

(gastroparesis:ti OR ‘delayed gastric emptying’:ti OR ‘gastric emptying delay’:ti)

AND

(’glucagon like peptide 1 receptor agonist*’:ab

OR ‘GLP-1 receptor agonist*’:ab

OR ‘GLP1 receptor agonist*’:ab

OR ‘incretin mimetic*’:ab

OR liraglutide:ab

OR semaglutide:ab

OR dulaglutide:ab

OR exenatide:ab

OR lixisenatide:ab

OR albiglutide:ab

OR taspoglutide:ab

OR efpeglenatide:ab)

AND [humans]/lim AND [english]/lim

**WEB OF SCIENCE**

“GLP-1 receptor agonist*” OR “GLP1 receptor agonist*” OR “glucagon like peptide 1 receptor agonist*” OR “incretin mimetic*” OR liraglutide OR semaglutide OR dulaglutide OR exenatide OR lixisenatide OR albiglutide OR taspoglutide OR efpeglenatide OR benaglutideANDgastroparesis OR “delayed gastric emptying” OR “gastric stasis” OR “impaired gastric motility” OR ileus OR “gastric emptying”

### Eligibility Criteria (PICOS Framework)

- Population: Adults (≥ 18 years) receiving GLP-1 receptor agonists for diabetes or weight reduction.
- Intervention: Treatment with any GLP-1RA (e.g., semaglutide, liraglutide, dulaglutide, exenatide, lixisenatide, or tirzepatide).
- Comparator: Not applicable
- Outcome: Gastroparesis including clinical features, diagnostic findings, management, and outcomes.
- Study design: case report, case series, case-control, cohort, randomised controlled trials

#### Exclusion criteria.

Animal studies, reviews, and editorials were excluded.

Studies not reporting gastroparesis clinical features, diagnostic evaluation, management and/or outcome were excluded. Studies that assessed gastric emptying using indirect physiological methods, such as paracetamol absorption tests and use of wireless motility capsule, were excluded unless they reported symptomatic gastroparesis confirmed by diagnostic evaluation.

### Study selection

**Fig 1:**
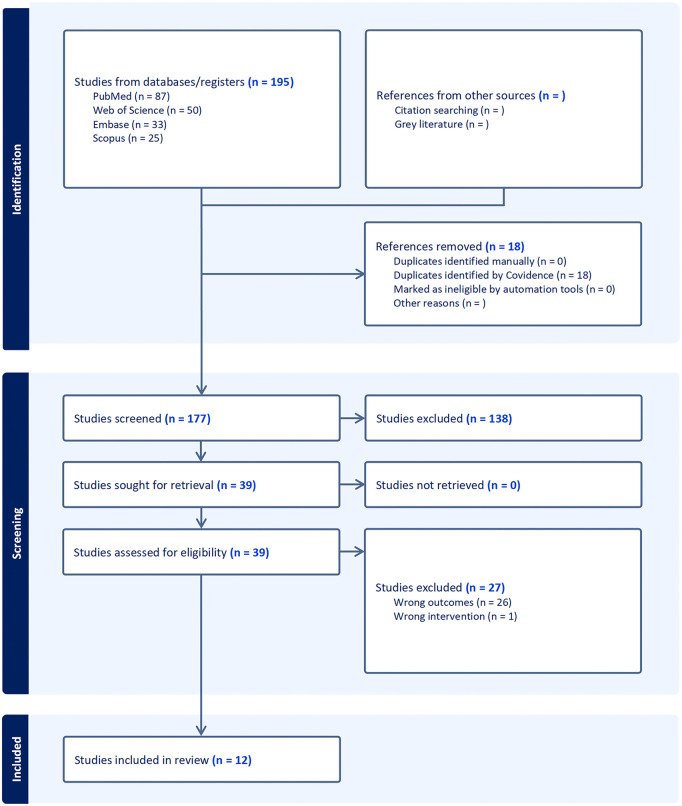
Prisma flow diagram.

Study selection was conducted independently by TO and NMBO, who screened titles and abstracts, assessed full-text articles, and applied the eligibility criteria. Discrepancies were resolved through discussion until consensus was reached. Reasons for exclusion were documented, and a PRISMA flow diagram was generated to illustrate the study selection process (Fig 1). The study selection process is presented in the PRISMA 2020 flow diagram. TO and NMBO extracted data from the included full-text articles; TO harmonised the extracted data and developed the summary tables.

### Data extraction

Data were extracted using a structured form covering the following domains:

(1) study characteristics (author, year, country, design)
(2) participant demographics and comorbidities
(3) GLP-1RA type, dose, and duration
(4) clinical presentation and symptom onset
(5) diagnostic methods and findings
(6) management strategies; and
(7) patient outcomes

### Quality assessment

All the studies included were case reports and case series. Thus, methodological quality was appraised using the Joanna Briggs Institute (JBI) Critical Appraisal Checklist for Case Reports (22). TO and NMBO, applied the JBI checklist for all included studies. Discrepancies were resolved through discussion until consensus was reached. Each study was rated across nine domains: clarity of patient demographics, clinical history and timeline, presenting features, diagnostic assessment, intervention description, post-intervention course, adverse event reporting, takeaway messages, and overall quality. Each domain was rated as low, high, or unclear risk.

### Data synthesis

A narrative synthesis was undertaken. Findings are summarised descriptively, in a table (Table 1) and narratively across predefined thematic domains, including patient demographics and comorbidities; type and dose of the GLP-1 receptor agonist implicated; clinical presentation; diagnostic approaches; management strategies; and outcomes. Consistencies, patterns, and gaps in the available evidence were identified and highlighted across studies. As all included studies provided relevant data across the predefined thematic domains, no studies were excluded from any part of the synthesis.

**Table 1:** Summary table of included studies showing study and patient characteristics, use of GLP-1RA, clinical presentation, diagnostic methods, management strategies and outcomes.

Study characteristics													
Study ID	Rai P. et al. 2018 [16]	Singhal R. et al. 2025 [17]	Almustanyir S. et al. 2020 [18]	Ishihara Y. et al. 2022 [19]	Chaudhry A. et al. 2024 [20]	Kalas et al. 2021 [21]	Kalas et al. 2021 [21]	Deirmenjian et al. 2024 [22]	Chahal 2023 [23]	Kalas et al. 2021 [24]	McGee et al. 2020 [25]	Chávez-Sánchez et al. 2024 [26]	Gomez P. et al. 2023 [27]
Study title	Liraglutide-induced Acute Gastroparesis.	Unmasking Semaglutide-Induced Gastroparesis: The Dangers of Rapid Dose Escalation in a Diabetic Patient.	Gastroparesis With the Initiation of Liraglutide: A Case Report.	Suspected Gastroparesis with Concurrent Gastroesophageal Reflux Disease Induced by Low-Dose Liraglutide.	Tendency of Semaglutide to Induce Gastroparesis: A Case Report.	Medication-Induced Gastroparesis: A Case Report (First patient)	Medication-Induced Gastroparesis: A Case Report (Second patient)	Gastroparesis Exacerbation by a GLP-1 Agonist	Slow Down and Think About It: Differentiating Causes of Gastroparesis in Patients with Diabetes	Is gastroparesis always irreversible in a diabetic?	Speed up a slow down: Recognizing GLP-1 gastroparesis and SGLT-2 euglycemic DKA	Severe gastroparesia associated with the use of GLP-1 receptor agonists for weight loss	I Miss Eating: Medication-Induced Gastroparesis from Semaglutide
Country	USA	Not stated	Saudi Arabia	Japan	USA	USA	USA	USA	USA	Not stated	USA	Not stated	USA
Study design	Case report	Case report	Case report	Case report	Case report	Case report (First patient)	Case report (Second patient)	Case report (conference abstract)	Case report (conference abstract)	Case report (conference abstract)	Case report (conference abstract)	case report (conference abstract)	Case report, (conference abstract)
**Patient demographics and comorbidities**													
Age, Sex	50-59 yrs, Male	40-49 yrs, Female	18-29 yrs, Female	≥70 yrs, Female	50-59 yrs, Female	50-59 yrs, Female	50-59 yrs, Female	40-49 yrs, Female	60-69 yrs, Male	57-60 yrs, Female	60-69 yrs, Male	60 - 69 yrs, Female	≥70 yrs, Male
Diabetes history	History of type 2 diabetes	History of type 2 diabetes	Short duration history of uncontrolled type 2 diabetes	History of type 2 diabetes	Not diabetic	A long-standing history of type 2 DM	A long-standing history of type 2 DM	Diabetic	Long standing history of Type 2 diabetes	A long-standing history of type 2 DM	A history of Type 2 DM. Duration not stated.	Not Diabetic	A long-standing history of type 2 DM
Blood glucose and HBA1c on admission	Well controlled DM	Well controlled DM	Poorly controlled DM	Poorly controlled DM	Not applicable	Well-controlled DM	Poorly controlled DM	Poorly controlled DM	Suboptimal or moderately controlled DM	Poorly controlled DM	HbA1C not stated	Not applicable	Well controlled DM. Prior to being on semaglutide his diabetes mellitus was uncontrolled
Obesity history	Not stated	Obese	Obese	Not stated	Obese	Not stated	Not stated	Obese	Not stated	Not stated	Not stated	Obese	Not stated
Gastroparesis history	No prior history of gastroparesis	Not stated	Not stated	Not stated	Not stated	Not stated	Not stated	Has diabetic gastroparesis. Had several gastroparesis flares in the past	Not stated	History suggestive of Diabetic gastroparesis prior to GLP-1RA use.	Previous history of resolved gastroparesis	Not stated	Not stated
Co-morbidities	Past medical history of hypertension, hyperlipidemia	History of hypertension, gastroesophageal reflux with a mild stricture and small hiatal hernia, and chronic constipation	Not stated	A history of hypertension	Medical history of depression, and obstructive sleep apnea	Not stated	Not stated	History of diabetic neuropathy, hyperlipidaemia	Not stated	Not stated	History of chronic pain	History of peptic ulcer	Not stated
Concomitant drugs	Not stated	Metformin, lisinopril, hydrochlorothiazide, sucralfate, omeprazole, and docusate	Insulin therapy for diabetes	Metformin, repaglinide, sitagliptin	Alprazolam, methocarbamol, desvenlafaxine, and bupropion.	Not stated	Not stated	Not stated	His medications included atorvastatin, fluvoxamine, losartan, metformin, pantoprazole,	Not stated	Medications included oxycodone, and dapagliflozin (a sodium-glucose co- transporter 2 [SGLT2] inhibitor).	History of use of NSAIDs	Not stated
**GLP-1RA indication, type, dose, duration**													
Indication for GLP-1RA	Diabetes management	Diabetes management	Diabetes and weight loss management	Diabetes management	Weight loss management	Diabetes management	Diabetes management	Diabetes management	Diabetes management	Diabetes management	Diabetes management	Weight management	Diabetes management
Type of GLP-1RA, dose and duration	Liraglutide: He had a recent start of Liraglutide 1.2 mg subcutaneously	Semaglutide: She was taking 2 mg semaglutide subcutaneously once weekly for diabetic management and had stopped taking it forone month. After visiting her primary care physician, she was advised to restart semaglutide at 0.5 mgsubcutaneously once weekly and then gradually titrate the dose upward until she reached her previousmaintenance dose. However, she restarted treatment directly at 2 mg subcutaneously once weekly withoutre-titration and continued to take this for the past four weeks.	Liraglutide: Recently, she was started on 3 mg of Liraglutide	Liraglutide: Had taken Liraglutide 0.6 mg for only four days	Semaglutide: Had taken semaglutide 0.5 mg for the preceding four months for weight loss	Semaglutide: Patient had started semaglutide subcutaneous injection weekly, 8 months before presentation	Dulaglutide: Patient started dulaglutide subcutaneous weekly injection approximately 15 months before presentation	Semaglutide: She was transitioned from dulaglutide to semaglutide after which she had a recurrent flare after two doses. Dose not stated	Semaglutide: His diabetes medications were changed from metformin 1000 mg daily and linagliptin 5 mg; to metformin 1500 mg daily and semaglutide 3 mg daily due to poor control. Semaglutide, was later increased to 7 mg daily and metformin dose decreased.	Dulaglutide: She started dulaglutide 1 year ago	Exenatide: Used 3 times of his intended dose of exenatide a day before presentation	Semaglutide: She was using semaglutide without the knowledge of her healthcare providers	Semaglutide: had used semaglutide for 7 months. Dose of semaglutide not stated
**Clinical presentation and symptom onset**													
Duration of drug use before onset of symptoms	Started using Liraglutide recently	About 1 week	Started using Liraglutide recently	One day	Three months	Symptoms developed approximately 1 month after starting semaglutide	About 3 months after starting dulaglutide	After two doses	He first noted a decrease in appetite and early satiety when placed on semaglutide 3 mg, 3 months before presentation. Symptoms worsened when dose was increased to 7 mg. Duration of drug use before onset of symptoms not stated	She started dulaglutide about 1 year before presentation. Symptoms actually started some years before presentation (i.e., before use of dulaglutide).	Symptoms began the following morning after taking an overdose of exenatide the previous day	Duration of use of semaglutide not stated	1 month
Symptoms on presentation	Presented with nausea, abdominal distension, and pain ofone-week duration. The patient initially reported symptoms of early satiety and excessive bloating, leadingto nausea and progressive abdominal distension. He then developed more acute, severe epigastric and left upper quadrant pain.	Nausea and non-bilious, non-bloody emesis for approximately three to four weeks. Left lower quadrantabdominal pain, subjective fevers, chills, and constipation.	Vomiting and upperabdominal discomfort for six days.	Sore throat, anorexia, nausea, andabdomen distention three days prior to presentation	Nausea and abdominal pain for three weeks	A 7-month history of postprandial epigastric pain, accompanied by fullness, bloating, and nausea. Multiple pharmacologics including proton pump inhibitors and antispasmodics (anticholinergics), provided no relief	Presented with abdominal bloating, nausea, and vomiting for the past year	One-month history of postprandial abdominal pain, nausea, vomiting, and significant unintentional weight loss with decreased oral intake. Her abdominal pain was sharp and was exacerbated with eating.	He presented with subacute progressive abdominal distension, nausea and vomiting after increase in dose of semaglutide	History of nausea, vomiting, early satiety, abdominal fullness and bloating of some years duration	Sudden onset severe lower abdominal pain, nausea and vomiting, and inability to keep food or drink down	Presented with abdominal pain and oral intolerance refractory to conventional management	Six months of nausea, vomiting, and abdominal pain.
Findings on general examination	Not stated	The patient was haemodynamically stable with normal temperature, heart rate, blood pressure, respiratory rate, and oxygen saturation on room air.	Found to be vitally stable.	Not stated	Not stated	Not stated	Not stated	Not stated	On exam, he was mildly tachycardic with dry mucus membranes.	Not stated	Otherwise normal	Not stated	Not stated
Findings on abdominal examination	Soft but mildly distended abdomen with mildtenderness to palpation in the epigastrium and left upper quadrant with faint bowel sounds and negativesuccussion splash.	Abdomen was soft, not distended, with normoactive bowel sounds. There was tenderness topalpation in the left greater than the right lower quadrants.	Abdominal exam displayed a distended abdomen withepigastric tenderness	Abdominal examination revealed that her abdomen was soft and non-tender but with decreased bowelsounds	The abdominal examination was significant for mild abdominal discomfort without rigidity,guarding, and rebound tenderness with normal bowel sounds.	Not stated	Not stated	On physical examination, her abdomen was diffusely tender to palpation.	Not stated	Not stated	Exam was notable for normal vitals with epigastric and periumbilical tenderness. Otherwise exam was normal	Not stated	Not stated
Other findings on examination	Not stated	Not remarkable	Not remarkable	Not stated	Not stated	Not stated	Not stated	Not stated	Not stated	Not stated	Not stated	Not stated	Not stated
**Diagnostic methods and findings**													
Result of Blood investigations	Normal liver function tests, normal lipase,normal CBC and chemistry	Elevated serum creatinine consistent with acute kidney injury (AKI) and mild leukocytosis.	Blood test results for creatinine,potassium, sodium, aspartate aminotransferase, and alanine aminotransferase were within normal levels	Liverfunction tests and complete blood counts were within normal limits	Autoimmune investigations were unremarkable.	Laboratory tests were unremarkable.	Not stated	Not stated	Mild hyperglycaemia; lipase within normal limits.	Not stated	High anion gap metabolic acidosis with elevated ketones, consistent with euglycaemic diabetic ketoacidosis (DKA).	Not stated	Not stated
Results of radiology investigations	Abdominal CT scan revealed markedly distended stomach with food/debris andnormal caliber duodenum without an obvious lesion.Abdominal x-ray showed a non-obstructive gas pattern with no intestinaldilatation.	Non-contrast CT of the abdomen and pelvis revealed multiple scattered diverticula and associated pericolic fat stranding, consistent with uncomplicated diverticulitis (colitis)	X-ray of abdomen displayed a non-obstructive gas pattern without intestinal dilatation. Abdominalcomputed tomography showed an isolated moderate-grade gastric distension, with smooth thin wall and noevidence of distal gastric, pyloric, or proximal duodenal obstructing masses	CT scan of her abdomen revealed fluid accumulation from the stomach to thehorizontal portion of the duodenum, but no signs of dilated small or large bowel, or evidence of mechanicalobstruction	CT scan of the abdomen showed no external obstruction and no other acute pathology	CT of the abdomen was unremarkable.	Not stated	Not stated	Abdominal CT showed a distendedstomach with food and fluid contents.	Not stated	Abdominal x-ray showed neither obstruction nor boweldistension.	Not stated	Not stated
Results of Endoscopic investigations	Shortly after admission, he underwent an upper endoscopy. Upper endoscopy showed no evidence of an obstructinglesion, tumor, or bezoar. The pylorus was patent and easily traversed. There was mild irritation in the gastric body, likely related to nasogastric tube trauma.	Not done (implied)	Esophagogastroduodenoscopy (EGD) exhibited no proof of an obstructing lesion.	Gastroscopy showed a patent pylorus along with reflux esophagitis (RE) of LosAngeles grade C.	Endoscopy revealed a normalesophagus and semisolid food in the fundus, hence, the procedure was aborted due to retained foodmaterial.	Prior upperand lower endoscopies showed no evidence of obstruction	Previousupper endoscopy and colonoscopy did not reveal any obstruction or other abnormalities	Not stated	Not stated	Prior upper and lower endoscopy showed no obstruction	Not stated	An upper digestive endoscopy was performed, diagnosing severe gastroparesis.	Upper endoscopy showed some retained food in the stomach but no evidence of obstruction
Results of Gastric motility tests carried out	Not done (implied)	Not done (implied)	Not done (implied)	Not done (implied)	A gastric emptying study, was not performed as the gastroenterologist had a highdegree of certainty of the diagnosis given symptoms and findings on EGD	A 4-hour scintigraphic gastric emptying study (GES)showed 24% retention of isotope in the stomach at 4 hourswhich indicates delayed gastric emptying (GE) as gastricresidual remaining at 4 hours should be < 10%	A 4-hour scintigraphic GES showed 35% reten-tion of isotope in the stomach at 4 hours, revealing delayed gastric emptying	Sheunderwent a gastric emptying study with the gastricamount retained as follows: 97% (at 1 hour), 88% (at 2hours), and 79% (at 4 hours).	Not done (implied)	A 4-hour GES was scheduled and showed 35% retention of isotope at 4 hours	Not done (implied)	Not stated	A scintigraphic gastricemptying study was completed and showed 33% gastric retention at 4 hours consistent with moderate gastroparesis
Results of other investigations done	Not stated	Blood cultures: Negative, Urine culture: Negative, Stool culture: Negative, Fecal lactoferrin: Negative, Stool calprotectin (mildly elevated → mild colonic inflammation) Urinalysis: unremarkable	Not stated	Not stated	Not stated	Biopsy was negative for Heliobacter pylori. A hepatobiliary iminodiacetic acid (HIDA) scan was also done in thepast which ruled out gallbladder dyskinesia, and an abdominal Doppler blood flow study ruled out median arcuate ligament syndrome	Not stated	Not stated	Urinalysis showed ketonuria.	Not stated	Urinalysis had ketones	Not stated	Not stated
**Management strategies**													
Treatment: Admitted or not admitted	He was admitted	She was admitted to the hospital for intractable nausea/vomiting as well as treatment of her left-sided colitis.	She was admitted	She was admitted	She was admitted	She was not admitted	She was not admitted	She was admitted	He was admitted	She was not admitted	Patient was admitted	Not stated	Not admitted (implied)
Treatment: Removing or addressing the underlying cause (Discontinuing the GLP-1RA)	Liraglutide was withheld	Semaglutide was discontinued	Intake of liraglutide was ceased	All diabetic medications were discontinued	Semaglutide was stopped	Semaglutide was held for 6 weeks with resolution of symptoms.	Dulaglutide was discontinued for 4 weeks with gradual resolution of symptoms	She was advised to never take aGLP-1 agonist again and should her symptoms not improvethe next step would be to pursue a gastric pacemaker.	Semaglutide was withheld	Dulaglutide was withheld for 4 weeks and symptoms resolved.	Exenatide and dapagliflozin werediscontinued, only at discharge.	Not stated	Initially, Semaglutide was not discontinued, and symptoms did not resolve. Eventually, Semaglutide was discontinued and symptoms resolved within one month
Treatment: Gastric decompression	A nasogastric tube was placed with over1 litre of fluids suctioned. This provided instant symptom relief but raised concern for gastric outletobstruction.	Gastric decompression not done/required (implied)	Gastric suctioning through a nasogastric tube was carried out	In view of severe nausea, a nasogastric tube was inserted, which aspirated 600mL of gastric contents	Gastric decompression was not done. She was able to tolerate a semisolid diet	Not stated	Not stated	She was made NPO	Not stated	Not stated	Not stated	Not stated	Gastric decompression not done/required (implied)
Pharmacologic Therapy	A brief course of antiemetics and metoclopramide, was given	She was initially started on ceftriaxone and metronidazole for the treatment of her colitis and potential UTI.Her nausea and vomiting were suspected to be semaglutide-induced. She was started on 10 mgmetoclopramide as needed every six hours and maintenance fluid.	The patient received a short course of antiemetics (ondansetron andmetoclopramide)	Intravenous metoclopramide 10 mg was administered.	The patient was started on IV ondansetron 4 mg every 6 hours as needed for nausea	Not stated	Not stated	She was initiated on scheduled metoclopramideGastroenterology recommended scheduled metoclopramideand erythromycin	Metoclopramide was initiated	Not stated	Patient was started on scheduled metoclopramide for delayed gastricemptying side effect of exenatide. The high AG acidosis, elevated serumand urine ketones, and mildly elevated serum glucose in an individual onSGLT-2 inhibitor, was concerning for euglycemic diabetic ketoacidosis(DKA). He was started on treatment for DKA with D5W1/2NS andinsulin drip. After AG closed he was switched to basal insulin.	She continued with supportive therapy	Pharmacologic therapy was given. Details not mentioned.
Nutritional support, dietary modification and hydration	Dietary modification,was done. Details not provided	Her acute kidney injury resolved on her second day of hospitalization, afterreceiving 2 L of fluids.	Consequently, gastric suctioning through a nasogastric tube was carried out to treat her condition and the intake of liraglutide was ceased.	Oral nutrition and all diabetic medications were discontinued.	She was able totolerate a semisolid diet.	Not stated	Not stated	She was initially made NPO, and started onperipheral fluids Gastroenterology later recommended dietary adjustments including small volume liquid calories. Her oral intake wasinsufficient and she opted for total peripheral nutrition until symptoms improved.	Was placed onintravenous fluids.	Not stated	He was started on treatment for DKA with D5W ½ NS (5% dextrose in half-normal saline) and insulin drip	Not stated	Dietary modification was done
Alternative drug(s) given in place of GLP-1RA	Not stated	She was advised to follow up with her primary care provider regarding her diabetic medicationregimen.	Not stated	Diabetes was managed with rapid-acting insulin from the day of admission.	Not stated	Not stated	Not stated	Not stated	Not stated	Not stated	He was discharged on basal insulin, prandial sliding scale insulin, and scheduled metoclopramide. Exenatide and dapagliflozin were discontinued at discharge.	Not stated	Not stated
**Patient outcomes**													
Outcome /Resolution of symptoms	symptoms completely resolved	Her symptoms resolved promptly after discontinuing the medication. After three doses of metoclopramide, her nausea and vomiting resolved. She was discharged after four days of hospitalization.	Discharged home after three days. Still doing well two weeks later.	On the third day of admission, nausea resolved, could tolerate oral nutrition. By 10th day, nausea and reflux symptoms had entirely disappeared	Patient reported significant improvement in symptoms at one-month follow-up with complete resolution of nausea.	Semaglutidewas held for 6 weeks with resolution of symptoms. A repeat GES was then performed and showed resolution of delayed GE.	Dulaglutide was discontinued for 4 weekswith gradual resolution of symptoms. A repeat GES was thenperformed and showed normalization of GE	Her hospital course was com-plicated by persistent symptoms of gastroparesis.Information was not provided on resolution of symptoms	The tachycardia resolved withintravenous fluids. His symptoms resolved within 24 hours of management, and patient was dischargedhome on metoclopramide	Dulaglutide was held for 4 weeks and symptoms resolved. A repeat GES showed 3% of isotope retained in the stomach at 4 hours, which is normal.	His gastrointestinal symptoms all rapidly improved with treatment of DKA andgastroparesis	She continued with supportive therapy and the symptoms resolved spontaneously	Dietary modifications and pharmacologic treatment were trialed initially, but his gastroparesis symptoms did not improve. In coordination with endocrinology, decision made to stop semaglutide and symptoms completely resolved within 1 month. A repeat gastric emptying study was performed and no longer showed delayed gastric emptying.

### Ethical considerations

This review analysed data from previously published studies and did not involve direct contact with human participants. Therefore, ethical approval and informed consent were not required.

## Results

A total of 12 case reports describing 13 patients with suspected or confirmed gastroparesis associated with GLP-1RAs met the inclusion criteria [16–27]. Eleven studies were individual case reports [16–20,22–27], while one was a case series that reported two cases [21]. Six journal articles were peer-reviewed [16–21] while the other six were published as conference abstracts [22–27]. Most of the studies originated from the United States [16,20–23,25,27], with additional reports from Japan [19] and Saudi Arabia [18]. (Table 1)

### Patient characteristics

Patients ranged from 18 to 74 years of age, with a predominance of female patients. Most individuals (11 of 13) had a documented history of type 2 diabetes mellitus, and long-standing in many cases and with varying degrees of glycaemic control. Two patients used GLP-1RAs exclusively for weight loss [20,26]. Three patients had a history suggestive of previous gastroparesis [22,24,25]. Obesity or overweight was commonly reported, consistent with the clinical indications for GLP-1RA therapy. Comorbidities included hypertension, hyperlipidaemia, gastroesophageal reflux disease, chronic constipation, depression, chronic pain, and peptic ulcer disease.(Table 1)

### GLP-1RA exposure

GLP-1RAs were prescribed primarily for diabetes management, although three cases involved weight-loss use. The implicated agents included semaglutide, dulaglutide, liraglutide, and exenatide. The duration of therapy prior to symptom onset varied, ranging from one day (after exenatide overdose) to three months. Three cases reported symptom onset following dose escalation [17,23,25], with one case occurring after a patient restarted semaglutide at her previous maximum dose without re-titration [17]. (Table 1)

### Temporal pattern of symptom onset

The interval between GLP-1RA exposure and symptom onset varied considerably across the included cases, ranging from within 24 hours to approximately 3 months after treatment initiation or re-exposure. Five patients developed symptoms within one week of GLP-1RA initiation, inappropriate re-initiation, or overdose [17–19,22,25]. Four patients developed symptoms between one and three months after treatment initiation or during dose escalation [20,21,27]. In one case, symptom onset was reported only as occurring shortly after recent initiation of liraglutide, precluding precise temporal classification [16]. In another patient, reduced appetite and early satiety were first noted after initiation of semaglutide and had been present for approximately three months before presentation, with symptoms worsening following dose escalation [23]. One patient had symptoms suggestive of gastroparesis before initiation of dulaglutide, with subsequent exacerbation after treatment, making the temporal relationship to GLP-1RA exposure difficult to classify [24].

### Clinical presentation

Across reports, patients presented symptoms characteristic of gastroparesis. The most common symptoms included nausea, vomiting, postprandial abdominal pain, abdominal distension or bloating, early satiety, and decreased oral intake [16–27]. Symptom severity varied, with several cases resulting in the inability to tolerate oral intake. One patient developed euglycaemic diabetic ketoacidosis, associated with concurrent exenatide overdose and an SGLT2 inhibitor [25].

Physical examination findings ranged from normal to evidence of dehydration, tachycardia, abdominal tenderness, distension, and reduced bowel sounds. Vital signs were often stable [17,25,28]. (Table 1)

### Diagnostic Evaluation

#### Laboratory findings.

Laboratory investigations commonly included assessment of glycaemic control, renal function, electrolytes, liver and pancreatic enzymes, and complete blood counts. Most laboratory tests were unremarkable. Isolated abnormalities included:

- High anion gap metabolic acidosis and elevated β-hydroxybutyrate in a patient with euglycaemic DKA [25].
- Acute kidney injury in one patient, which resolved with hydration [17], and
- Mild inflammatory findings associated with colitis in another case [17].

Routine chemistry, liver enzymes, and complete blood counts were generally within normal limits. (Table 1)

#### Radiologic and endoscopic findings.

Cross-sectional imaging (CT or abdominal radiography) frequently demonstrated gastric distension [17,19,20,23] and no evidence of mechanical obstruction [18–20,25], a consistent pattern across cases. Upper gastrointestinal endoscopy frequently revealed retained gastric contents [20,27] with no evidence of mechanical obstruction [16,18,21,24,27]. In one case, reflux esophagitis was observed. (Table 1)

#### Gastric emptying studies.

Six patients underwent scintigraphic gastric emptying studies (GES) [20–22,24,27]. All demonstrated delayed gastric emptying, with 4-hour retention ranging from 24% to 88%, indicative of mild to severe gastroparesis. In cases where repeat GES was performed after discontinuation of the GLP-1RA, gastric emptying normalized [21,24,27]. (Table 1)

### Management strategies

#### Withdrawal of GLP-1RA.

Discontinuation of the implicated GLP-1RA represented the cornerstone of management [16–27]. In nearly all cases, symptom improvement occurred after the GLP-1RA was withdrawn [16–20,22–27]. One patient had persistent symptoms until semaglutide was eventually discontinued after initial treatment failure [27]. (Table 1)

#### Supportive and pharmacologic therapy.

Most patients received antiemetics, prokinetic agents (particularly metoclopramide), and intravenous fluids for hydration. In the patient presenting with euglycaemic DKA, insulin therapy and dextrose-containing fluids were administered [25]. (Table 1)

#### Gastric decompression and nutritional support.

A minority of patients required nasogastric tube decompression, yielding immediate symptomatic relief and confirming delayed gastric emptying [16,18,19]. Nutritional support included temporary NPO status, gradual diet advancement, liquid or small-volume meals, and, in one case, peripheral parenteral nutrition due to poor oral intake [22]. (Table 1)

### Outcomes

Clinical outcomes were favourable in nearly all cases [16–20,22–27]. Symptom resolution occurred after GLP-1RA withdrawal. Patients who underwent repeat gastric emptying studies demonstrated complete normalization of gastric motility. Hospital stays ranged from three to ten days. No mortality or long-term complications were reported. Only one patient experienced a more prolonged or complicated course, and this patient had had several flares of diabetic gastroparesis preceding the use of semaglutide [21]. (Table 1)

### Risk of bias analysis

Most included studies demonstrated low risk of bias across the major clinical reporting domains, with clear descriptions of patient characteristics, clinical presentation, diagnostic investigations, and interventions. However, several reports provided limited information on post-intervention clinical course and adverse events, leading to *unclear* ratings in these domains. One study showed high risk of bias across multiple domains due to insufficient reporting of patient history, diagnostic methods, and clinical outcomes [26]. Conference abstracts also featured inherent reporting limitations [24,26].

Overall, the majority of case reports (10 out of 12) were judged to have low overall risk of bias (Table 2)

**Table 2:** Risk of bias using the Joanna Briggs Institute (JBI) critical appraisal checklist for case reports.

Study ID	Were patient’s demographic characteristics clearly described?	Was the patient’s history clearly described and presented as a timeline?	Was the current clinical condition of the patient on presentation clearly described?	Were diagnostic tests or assessment methods and the results clearly described?	Was the intervention(s) or treatment procedure(s) clearly described?	Was the post-intervention clinical condition clearly described?	Were adverse events (harms) or unanticipated events identified and described?	Does the case report provide takeaway lessons?	Overall quality judgment
Deirmenjian 2024	low	low	low	low	low	unclear	low	high	**low**
Gomez 2023	low	low	unclear	low	low	low	high	low	**low**
Chahal 2023	low	low	low	low	low	low	unclear	low	**low**
Kalas 2021	low	low	low	low	unclear	low	unclear	low	**low**
Kalas 2021	low	low	low	low	low	low	unclear	low	**low**
McGee 2020	low	low	low	low	low	low	unclear	low	**low**
Rai 2018	low	low	low	low	unclear	unclear	low	low	**unclear**
Singhal 2025	low	low	low	low	low	low	low	low	**low**
Almustanyir 2020	low	low	low	low	low	low	unclear	low	**low**
Ishihara 2022	low	low	low	low	low	low	low	low	**low**
Chaudhry 2024	low	low	low	low	low	low	unclear	low	**low**
Chávez-Sánchez 2024	low	high	high	high	high	unclear	unclear	high	**high**

## Discussion

This systematic review synthesizes published case-based evidence on gastroparesis induced or exacerbated by glucagon-like peptide-1 receptor agonists (GLP-1RAs), highlighting clinical features, diagnostic approaches, management strategies, and patient outcomes. Across the included studies, a consistent temporal association emerged between GLP-1RA exposure—particularly semaglutide, liraglutide, dulaglutide, and exenatide—and the onset or worsening of gastroparesis symptoms, with symptom resolution in most cases following drug discontinuation.

### Patient demographics and comorbidities

Most reported cases of GLP-1RA-associated gastroparesis involved middle-aged to older adults, although one case was reported in an adolescent female. GLP-1RA-associated gastroparesis was most commonly reported in individuals with type 2 diabetes mellitus. This may imply that GLP-1RA-associated gastroparesis is more common among diabetics than among non-diabetics, which could be explained by underlying diabetic neuropathy [28]. We cannot however causally imply that GLP-1RA-associated gastroparesis is more common among people with diabetes due to the descriptive rather than analytical nature of the included studies. Future experimental and analytical studies will be needed to establish this association.

In our review, three of the 13 cases had evidence of pre-existing or subclinical gastroparesis, and all three were diabetics. In these cases, long-standing symptoms suggestive of diabetic gastroparesis preceded GLP-1RA initiation, indicating that the medication likely acted as a precipitating or exacerbating factor rather than a sole cause [28]. Clinicians should, therefore, carefully screen for a history suggestive of pre-existing gastroparesis before initiating GLP-1RAs in patients with diabetes. It is important to note however that the majority of diabetic patients included in this review had no prior history suggestive of gastric motility delay.

Two cases of GLP-1RA-associated gastroparesis in this review, were observed in non-diabetic patients. This finding suggests that GLP-1RA-associated gastroparesis can also occur in non-diabetics and is not exclusively related to patients with diabetes or diabetic autonomic neuropathy. Some RCTs have also shown that GLP-1RA induced gastroparesis can also occur in non-diabetic populations. For example, an RCT conducted among non-diabetic participants demonstrated that treatment with liraglutide significantly prolonged gastric emptying scintigraphy half-time compared with placebo [29].

### Type of GLP-1RA implicated and dose

Across the included reports, long-acting GLP-1 receptor agonists—particularly semaglutide (seven out of 13 cases), followed by liraglutide—were most commonly implicated. Only one case involved a short-acting agent (exenatide), and in this case, gastroparesis occurred following accidental administration of a dose three times higher than prescribed. While prior literature suggests that short-acting GLP-1RAs are more strongly associated with delayed gastric emptying due to delay in tachyphylaxis setting in with the short acting formulations, we observed a different pattern in this systematic review [30,31]. Our observation that gastroparesis occurred more commonly with long acting GLP-1RA should be interpreted with caution, as this may only reflect the wider contemporary use of long acting GLP-1RAs compared with short-acting formulations. Long acting GLP-1RAs have the advantage of better compliance and ease of use than the short acting formulations and have shown better glycemic and weight control [32,33]. As clinicians prescribe long acting GLP-1RAs, they should be aware of the risk of Gastroparesis.

In this review, four cases of gastroparesis developed following dose escalation, rapid titration contrary to prescribing recommendations, or unsupervised use of GLP-1RAs. These findings underscore the importance of gradual, clinician-guided dose titration when initiating or escalating GLP-1RA therapy. One case occurred on switching from one type of GLP-1RA (dulaglutide) to another (semaglutide). This also calls for awareness of potential adverse effects when changing from one type of GLP-1RA to another.

### Temporal pattern of symptom onset

The temporal pattern observed in this review indicates that GLP-1RA-associated gastroparesis can occur at different stages of treatment rather than being confined to the initiation phase. Although most patients developed symptoms within one week of GLP-1RA initiation, inappropriate re-initiation, or overdose, several others presented after one to three months of therapy, often during or following dose escalation. This variability suggests that the development of gastroparesis may be influenced by multiple factors, including individual susceptibility, cumulative drug exposure, and treatment-related factors such as dose escalation or inappropriate re-initiation after treatment interruption. Clinicians should therefore remain vigilant for persistent upper gastrointestinal symptoms throughout the course of GLP-1RA therapy and not limit suspicion to the immediate/early treatment period. The occurrence of symptoms after dose escalation further underscores the importance of gradual dose titration in accordance with prescribing recommendations and careful monitoring of patients who develop persistent nausea, vomiting, early satiety, abdominal bloating, or abdominal pain.

### Clinical presentation

Across studies, patients presented with upper gastrointestinal symptoms dominated by nausea, vomiting, and abdominal pain, with symptom duration ranging from days to over one year. Postprandial epigastric pain, early satiety, fullness, bloating, and progressive abdominal distension were frequently reported and often worsened with eating. More severe cases involved oral intolerance with inability to tolerate food or fluids, occasionally accompanied by significant weight loss. These symptoms are not different from those documented in literature as symptoms of gastroparesis and symptoms of diabetic gastroparesis [34,35]. However, these symptoms are not specific to gastroparesis and overlap considerably with those of functional dyspepsia, making differentiation on the basis of symptoms alone difficult. Emerging evidence suggests that functional dyspepsia and gastroparesis may represent overlapping disorders or, in some patients, a spectrum of gastroduodenal neuromuscular dysfunction rather than entirely distinct disease entities. Consequently, symptoms should be interpreted within the overall clinical context, including their temporal relationship with GLP-1RA exposure, symptom severity and persistence, and the presence of features suggestive of delayed gastric emptying [36,37]. Recent expert commentary has further argued that differentiating gastroparesis from functional dyspepsia on the basis of gastric emptying alone may no longer be sufficient, suggesting that these conditions are better viewed as overlapping syndromes rather than entirely distinct disease entities [38].

In this review, chronic presentations were often refractory to standard therapies, while acute cases were marked by sudden symptom onset and rapid progression. Upper gastrointestinal symptoms in patients receiving GLP-1RAs should not be routinely dismissed as benign or tolerable adverse effects. In some cases, severe or persistent symptoms may reflect underlying gastroparesis and warrant appropriate diagnostic evaluation and management. Clinicians should therefore maintain a high index of suspicion, while avoiding undue alarm in cases of mild, self-limiting nausea or vomiting.

Our findings are consistent with emerging observational evidence suggesting that GLP-1RAs are associated with an increased risk of gastroparesis. In a large retrospective cohort study of over 55,000 individuals with obesity but without type 2 diabetes, semaglutide use was associated with a significantly higher risk of gastroparesis than bupropion-naltrexone or sleeve gastrectomy, although the absolute incidence remained low (6.5 per 1000 person-years). These findings complement the present review by demonstrating that, while GLP-1RA-induced gastroparesis is uncommon at the population level, clinically significant cases do occur and warrant prompt recognition [39].

### Diagnostic approaches

Laboratory investigations were variably reported across cases and included metabolic, renal, haematological, pancreatic, hepatic, acid–base, and immunological assessments. All diabetic patients had blood glucose and/or HBA1c tests done. Some investigations were requested based on other concurrent presenting features rather than gastroparesis itself. Laboratory tests were largely unremarkable, with exception of a few cases, e.g., a case of acute kidney injury which showed elevated creatinine, and a high white blood cell count reported in a case of concurrent colitis. It is important clinicians request baseline laboratory tests including serum glucose, renal function tests, serum electrolytes and any other necessary tests to rule out poor glucose control, complications from fluid and electrolyte derangements, and any concurrent conditions [7].

Diagnostic evaluation of gastroparesis varied across the included studies. In most cases, radiological investigations and endoscopy were used to demonstrate gastric distension or retained gastric contents and to exclude mechanical obstruction. When interpreted alongside the temporal relationship between symptom onset and GLP-1RA exposure, these findings supported a suspected diagnosis of GLP-1RA-associated gastroparesis. We observed in this review that Gastric emptying scintigraphy (GES) was performed only among patients with chronic or long-standing symptoms. In most of these cases, long standing symptoms were suggestive of pre-existing gastroparesis or gastric motility disorders and GES was done to confirm delayed gastric emptying and also to document reversibility after drug withdrawal.

### Management strategies

Across all studies, the cornerstone of management was discontinuation of the offending GLP-1RA, which consistently resulted in symptom improvement or resolution. The cases that were admitted to the hospital were mostly cases with acute presentation and severe symptoms including dehydration, inability to tolerate orally, marked abdominal pain and vomiting. Supportive measures—including nil per os (NPO), intravenous fluids, electrolyte correction, and dietary modification—were commonly employed, particularly in hospitalized patients. Prokinetic agents, most frequently metoclopramide, were used in several cases, sometimes in combination with antiemetics. While pharmacologic therapy provided symptomatic relief, definitive improvement typically followed drug cessation. This highlights the importance of addressing the underlying cause, while providing supportive and symptomatic relief.

Severe cases required nasogastric decompression, especially when marked gastric distension or intractable vomiting was present. Rarely, GLP-1RA-induced gastroparesis occurred alongside serious metabolic complications, such as euglycemic diabetic ketoacidosis in patients on concurrent SGLT-2 inhibitors, requiring multidisciplinary inpatient management. The management of patients in this review were in line with laid down principles [7,8]. In all cases of gastroparesis in this study, invasive interventions were far reaching and not warranted.

### Outcomes and reversibility

Overall, patient outcomes were favorable. Most patients experienced complete symptom resolution within days to weeks of GLP-1RA discontinuation. Importantly, repeat GES in several cases demonstrated normalization of gastric emptying after discontinuation of GLP-1RAs, providing objective evidence that GLP-1RA-associated gastroparesis is frequently reversible. Of note is a particular case managed with supportive and symptomatic treatment but without cessation of GLP-1RA, the presenting symptoms persisted until the GLP-1RA was removed and the patient’s symptoms completely resolved within one month. Clinicians should approach management of GLP-1RA induced gastroparesis with the awareness of the need to stop the offending agent and consider other means of diabetes or weight management.

In only two of the studies included in this review, was the alternative drug used to replace the GLP-1RA for glucose control stated in the case report. In these two studies, insulin was used in place of GLP-1RA while the patients were on admission. Many patients were referred to the endocrinologist or their physician for proper management of their diabetes, after being treated for gastroparesis.

In one case in this systematic review, it was reported that the patient’s hospital course was complicated by persistent symptoms of gastroparesis, and there was no mention of resolution of her symptoms [22]. The patient involved was a middle-aged known diabetic female, who had prior history of diabetic gastroparesis. She had had several flares of gastroparesis in the past, and she had a severe case of gastroparesis on transition to semaglutide, from using dulaglutide, even after only two doses. Despite discontinuation of semaglutide, her symptoms persisted during hospitalisation, and gastric electrical stimulation was considered as a potential therapeutic option should symptoms fail to improve. She was also advised to permanently avoid further use of GLP-1 receptor agonists.

In contrast to other cases in this review, which generally demonstrated symptom resolution following withdrawal of the offending agent and supportive management, the prolonged and refractory course observed in this patient likely reflects underlying diabetic autonomic neuropathy with pre-existing gastroparesis. In this context, GLP-1RA exposure appears to have acted as a potent exacerbating factor rather than a primary cause. The severity of her presentation was underscored by profound weight loss, marked gastric retention on gastric emptying scintigraphy (79% retention at 4 hours), and inability to tolerate oral intake, necessitating parenteral nutrition.

While most cases of GLP-1RA-associated gastroparesis in this review were reversible, this case highlights the need for heightened caution when prescribing GLP-1RAs to patients with a known history of gastroparesis or features suggestive of diabetic autonomic neuropathy.

### Distinguishing drug-induced gastroparesis from exacerbation of underlying diabetic gastroparesis

The cases included in this review suggest that GLP-1RA-associated gastroparesis is not a uniform clinical entity but likely comprises both de novo drug-induced gastroparesis and exacerbation of pre-existing diabetic gastroparesis. Most reported cases were consistent with probable de novo gastroparesis, as patients had no documented history of gastroparesis before GLP-1RA initiation, developed symptoms shortly after starting therapy, following dose escalation, or after inappropriate re-initiation, and experienced symptom resolution after discontinuation of the offending agent [16–21,26,27]. In contrast, a smaller number of reports were more consistent with exacerbation of underlying diabetic gastroparesis. These included patients with documented diabetic gastroparesis and previous disease flares, a history of symptoms suggestive of gastroparesis predating GLP-1RA therapy, or previously resolved gastroparesis that recurred after GLP-1RA exposure [22,24,25]. However, in several patients with longstanding diabetes, baseline gastric emptying studies were unavailable, making it difficult to determine whether GLP-1RA therapy induced de novo gastroparesis or unmasked previously unrecognised diabetic gastroparesis. This distinction is clinically important because diabetes itself is a well-established cause of delayed gastric emptying, and GLP-1RAs may either induce gastric dysmotility in susceptible individuals or exacerbate existing impairment.

### Clinical implications and future directions

Based on the findings of this systematic review, several clinical recommendations can be made to guide safe prescribing of GLP-1RA and the management of GLP-1RA induced gastroparesis. Prior to initiation, clinicians should assess for baseline gastrointestinal symptoms and any history suggestive of gastroparesis, particularly in patients with long-standing or poorly controlled diabetes. GLP-1RAs should be started at the lowest recommended dose and titrated gradually, avoiding rapid dose escalation or abrupt re-initiation at high doses following treatment interruption. New or worsening gastrointestinal symptoms – including persistent nausea, vomiting, early satiety, abdominal bloating, or unexplained weight loss – after GLP-1RA initiation should prompt early consideration of medication-induced gastroparesis. In suspected cases, timely discontinuation of the GLP-1RA should be prioritized.

Gastric emptying studies should be used selectively, with greatest utility in patients with chronic or persistent symptoms, diagnostic uncertainty, or when confirmation is required to guide long-term management. In other settings, radiologic or endoscopic evidence excluding mechanical obstruction, together with symptoms of gastroparesis following medication exposure, is often sufficient to support the diagnosis. Supportive measures, including short-term use of prokinetic agents, antiemetics, dietary modification, hydration, and gastric decompression, may be necessary in moderate to severe cases but should not delay withdrawal of the offending agent. Finally, patients with known diabetic gastroparesis or autonomic neuropathy should be treated with heightened caution, and alternative therapies should be considered when available.

### Strengths, Limitations and Future Direction

This systematic review has several key strengths. It provides a comprehensive and structured synthesis of the existing clinical literature on GLP-1 receptor agonist–associated gastroparesis, an adverse effect of growing relevance with the widespread use of these agents. By collating evidence from multiple reports, the review captures a broad spectrum of clinical presentations, ranging from acute to chronic disease, in both diabetic and non-diabetic populations.

A major strength of this study is the detailed patient-level characterization of symptoms, timing of onset, diagnostic methods, management approaches, and outcomes. This granular approach allows for clearer identification of recurring clinical patterns and temporal relationships between GLP-1RA exposure and symptom development. In addition, the inclusion of cases with objective diagnostic confirmation and documented reversibility of delayed gastric emptying strengthens the overall interpretability of the findings.

This systematic review has important limitations that should be considered when interpreting the findings. The available evidence is derived almost exclusively from case reports and conference abstracts, which are inherently subject to publication bias, incomplete reporting, and limited generalizability. Many of the included studies were conference abstracts, which often provided limited clinical detail. Consequently, information on patient characteristics, baseline comorbidities, medication history, diagnostic evaluation, management strategies, duration of follow-up, and clinical outcomes was incomplete or inconsistently reported across some cases. This may have affected our ability to comprehensively compare cases, distinguish de novo GLP-1RA-induced gastroparesis from exacerbation of pre-existing gastroparesis, and assess the consistency of management approaches and patient outcomes. As a result, the true incidence and risk magnitude of GLP-1RA-induced gastroparesis cannot be determined from the current literature. Additionally, the predominance of single-patient reports limits the ability to perform quantitative synthesis or risk stratification. Future research should focus on prospective studies to estimate incidence, identify high-risk populations, and clarify dose-response relationships.

### Practical clinical approach

**Fig 2:**
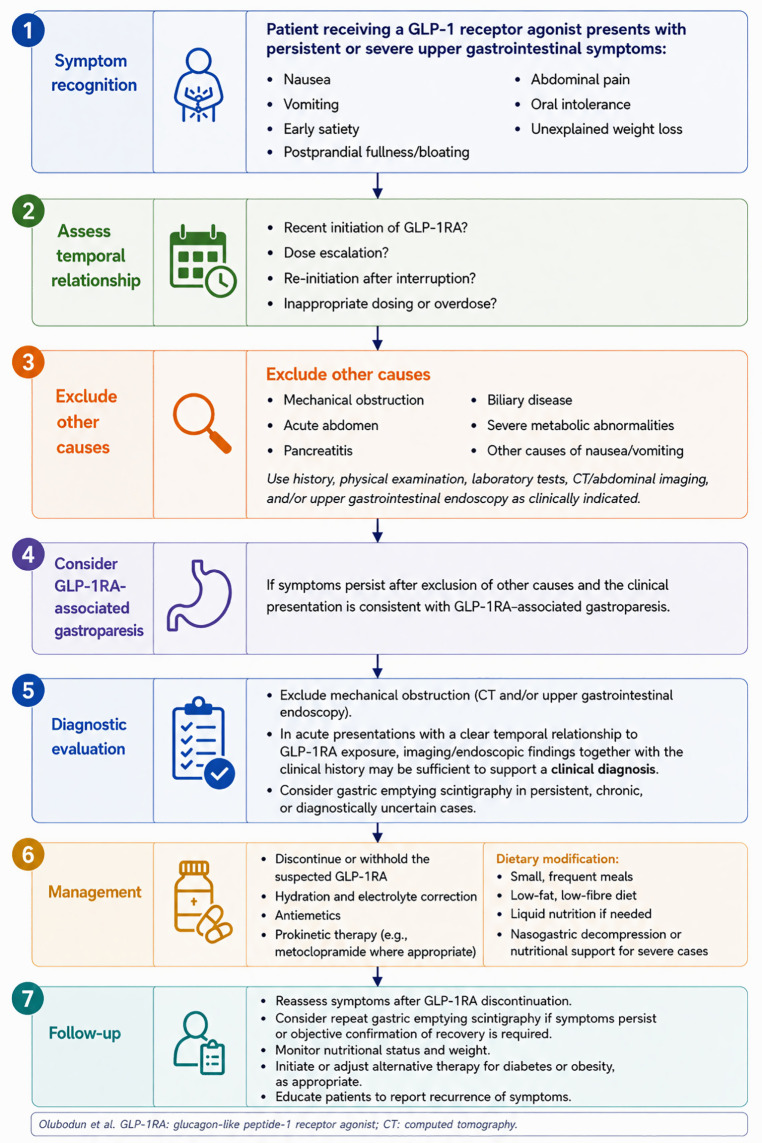
Proposed clinical approach to suspected glucagon-like peptide-1 receptor agonist (GLP-1RA)-associated gastroparesis (Olubodun et al.).

Based on the findings of this systematic review, Fig 2 presents a proposed practical clinical algorithm to assist clinicians in the recognition, diagnosis, and management of suspected GLP-1RA-associated gastroparesis. The algorithm emphasizes early recognition of persistent upper gastrointestinal symptoms, assessment of their temporal relationship to GLP-1RA therapy, exclusion of alternative causes including mechanical obstruction, appropriate diagnostic evaluation, prompt discontinuation of the suspected GLP-1RA when clinically indicated, supportive management, and follow-up. Although derived from case-based evidence and intended as a clinical aid rather than a formal guideline, the algorithm provides a pragmatic approach that may facilitate timely diagnosis and management in routine practice.

The algorithm, derived from the evidence synthesized in this systematic review, provides a practical approach to the recognition, diagnostic evaluation, management, and follow-up of patients with suspected GLP-1RA-associated gastroparesis. It is intended as a clinical aid rather than a formal clinical guideline.

## Conclusion

GLP-1 receptor agonists are increasingly prescribed for the management of diabetes and obesity, but they may induce or exacerbate gastroparesis through their pharmacologic effect on gastric motility. This systematic review demonstrates a consistent temporal relationship between GLP-1RA exposure and gastroparesis symptoms, with reversibility following drug discontinuation. Both patients with, and without diabetes may be affected, particularly after dose escalation or inappropriate use. Clinician awareness of this adverse effect is essential to ensure early recognition, appropriate management, and avoidance of unnecessary diagnostic procedures.

### Registration and protocol

The review was not registered. A protocol was not published but it can be obtained from the corresponding author, on request.

## Supporting information

S1 Table PRISMA 2020 Checklist. (DOCX)

## References

[1] Mariam Z, Niazi SK. Glucagon-like peptide agonists: A prospective review. Endocrinol Diabetes Metab. 2024;7(1):e462. doi: 10.1002/edm2.462 38093651 PMC10782143

[2] Westermeier F, Fisman EZ. Correction: Glucagon-like peptide-1 receptor agonists (GLP-1RAs) and cardiometabolic protection: historical development and future challenges. Cardiovasc Diabetol. 2025;24(1):118. doi: 10.1186/s12933-025-02647-2 40075386 PMC11905680

[3] Zhao X, Wang M, Wen Z, Lu Z, Cui L, Fu C, et al. GLP-1 Receptor Agonists: Beyond Their Pancreatic Effects. Front Endocrinol. 2021;12:721135. doi: 10.3389/fendo.2021.721135 PMC8419463 34497589

[4] Sharma D, Verma S, Vaidya S, Kalia K, Tiwari V. Recent updates on GLP-1 agonists: current advancements & challenges. BioMed Pharmacother. 2018;108:952–62. doi: 10.1016/j.biopha.2018.08.088 30372907

[5] Nauck MA, Meier JJ. Pharmacokinetics and pharmacodynamics of GLP-1 receptor agonists. Best Pract Res Clin Endocrinol Metab. 2019;33(2):101–16. doi: 10.1016/j.beem.2019.02.002

[6] Drucker DJ. Mechanisms of Action and Therapeutic Application of Glucagon-like Peptide-1. Cell Metab. 2018;27(4):740–56. doi: 10.1016/j.cmet.2018.03.001 29617641

[7] Camilleri M, Parkman HP, Shafi MA, Abell TL, Gerson L, American College of Gastroenterology. Clinical guideline: management of gastroparesis. Am J Gastroenterol. 2013;108(1):18–37; quiz 38. doi: 10.1038/ajg.2012.373 23147521 PMC3722580

[8] Parkman HP, Hasler WL, Fisher RS, American Gastroenterological Association. American Gastroenterological Association technical review on the diagnosis and treatment of gastroparesis. Gastroenterology. 2004;127(5):1592–622. doi: 10.1053/j.gastro.2004.09.055 15521026

[9] Joy B, Ramsamooj A, Ibrahim S, Wen J, Hsu N, Frezza E. Exploring the rates of Gastrointestinal adverse effects among five GLP-1 receptor agonists: A systematic review and Meta-Analysis of randomized controlled trials. Endocrine. 2026;91(1):23. doi: 10.1007/s12020-025-04494-3 41489846

[10] Zhang Z, Zhang Q, Tan Y, Chen Y, Zhou X, Liu S, et al. GLP-1RAs caused gastrointestinal adverse reactions of drug withdrawal: a system review and network meta-analysis. Front Endocrinol (Lausanne). 2023;14:1149328. doi: 10.3389/fendo.2023.1149328 37484944 PMC10359616

[11] Chiang C-H, Jaroenlapnopparat A, Colak SC, Yu C-C, Xanthavanij N, Wang T-H, et al. Glucagon-Like Peptide-1 Receptor Agonists and Gastrointestinal Adverse Events: A Systematic Review and Meta-Analysis. Gastroenterology. 2025;169(6):1268–81. doi: 10.1053/j.gastro.2025.06.003 40499738

[12] Baig MU, Piazza A, Lahooti A, Johnson KE, Rangwani S, Gouda Z, et al. Glucagon-like peptide-1 receptor agonist use and the risk of residual gastric contents and aspiration in patients undergoing GI endoscopy: a systematic review and a meta-analysis. Gastrointest Endosc. 2025;101(4):762–771.e13. doi: 10.1016/j.gie.2024.12.019 39694296

[13] Abdulraheem A, Abujaber B, Ayers L, Al-Zureikat Q, Bashiri K, Almhanni G, et al. Impact of GLP-1 receptor agonists on upper gastrointestinal endoscopy: an updated systematic review and meta-analysis. Surg Endosc. 2025;39(8):5135–51. doi: 10.1007/s00464-025-11962-4 40634731

[14] Elmati PR, Jagirdhar GSK, Qasba RK, Perez A, Qasba RK, Bains Y, et al. GLP-1 Agonists and the Risk of Pulmonary Aspiration during Elective Upper Endoscopy: A Systematic Review and Meta-analysis. Open Respir Med J. 2025;19. doi: 10.2174/0118743064372550250603061720 41036293 PMC12481593

[15] JBI Global. Checklist for Case Reports. https://jbi.global/sites/default/files/2020-08/Checklist_for_Case_Reports.pdf Accessed 2023 October 1.

[16] Rai P, Madi MY, Dickstein A. Liraglutide-induced acute gastroparesis. Cureus. 2018;10(12). 10.7759/cureus.3791 PMC6402745 30868005

[17] Singhal R, Sachdeva D, Wortman I I K, Lall R. Unmasking semaglutide-induced gastroparesis: the dangers of rapid dose escalation in a diabetic patient. Cureus. 2025;17(9):e91679. 10.7759/cureus.91679 PMC12497442 41054677

[18] Almustanyir S, Alhabeeb H, AlHusseini N, Al Thow M. Gastroparesis With the Initiation of Liraglutide: A Case Report. Cureus. 2020;12(11):e11735. doi: 10.7759/cureus.11735 PMC7773310 33403167

[19] Ishihara Y, Nishiguchi S, Branch J, Tanaka E. Suspected gastroparesis with concurrent gastroesophageal reflux disease induced by low-dose liraglutide. Cureus. 2022;14(7). 10.7759/cureus.26916 PMC9376210 35983392

[20] Chaudhry A, Gabriel B, Noor J, Jawad S, Challa SR. Tendency of semaglutide to induce gastroparesis: A case report. Cureus. 2024;16(1):e52564. doi: 10.7759/cureus.52564 PMC10874596 38371020

[21] Kalas MA, Galura GM, McCallum RW. Medication-Induced Gastroparesis: A Case Report. J Investig Med High Impact Case Rep. 2021;9. doi: 10.1177/23247096211051919 34663102 PMC8529310

[22] Deirmenjian JM, Dagher C. 6486 Gastroparesis Exacerbation by a GLP-1 Agonist. Journal of the Endocrine Society. 2024;8(Supplement_1). doi: 10.1210/jendso/bvae163.780

[23] Chahal N, Zarka J, Simonson M. Slow down and think about it: differentiating causes of gastroparesis in patients with diabetes. Injournal of General Internal Medicine. 2023;38:S533–S533.

[24] Kalas M, Galura GM, Sarosiek I, McCallum R. Is gastroparesis always irreversible in a diabetic?: 275. Journal of Investigative Medicine. 2021;69(2):527–8.

[25] McGee C, Lessing J. Speed up a slow down: Recognizing GLP-1 gastroparesis and SGLT-2 euglycemic DKA. Journal of General Internal Medicine. One New York Plaza, Suite 4600, New York, NY, United States: Springer. 2020:S565–S565.

[26] Chávez-Sánchez SA, Cedrón-Cheng HG. Severe gastroparesia associated with the use of GLP-1 receptor agonists for weight loss. Rev Gastroenterol Peru. 2024;44(1):71–4. 38734915

[27] Gomez P, Gutierrez VA, Narang N, Goldberg A. S4332 I Miss Eating: Medication-Induced Gastroparesis from Semaglutide. Am J Gastroenterol. 2023;118(10S):S2726–S2726. doi: 10.14309/01.ajg.0000966968.44182.37

[28] Bortolotti M. Gastroparesis, a diabetic complication causing further, even serious, complications: How to prevent its worsening? World J Gastroenterol. 2025;31(23):104932. doi: 10.3748/wjg.v31.i23.104932 40575340 PMC12188790

[29] Maselli D, Atieh J, Clark MM, Eckert D, Taylor A, Carlson P, et al. Effects of liraglutide on gastrointestinal functions and weight in obesity: A randomized clinical and pharmacogenomic trial. Obesity (Silver Spring). 2022;30(8):1608–20. doi: 10.1002/oby.23481 35894080 PMC9335902

[30] Madsbad S. Review of head-to-head comparisons of glucagon-like peptide-1 receptor agonists. Diabetes Obes Metab. 2016;18(4):317–32. doi: 10.1111/dom.12596 26511102 PMC5064617

[31] Quast DR, Schenker N, Menge BA, Nauck MA, Kapitza C, Meier JJ. Effects of lixisenatide versus liraglutide (short- and long-acting GLP-1 receptor agonists) on esophageal and gastric function in patients with type 2 diabetes. Diabetes Care. 2020;43(9):2137–45. doi: 10.2337/dc20-0720 32647054

[32] Yuan X, Gao Z, Hao Z, Ma H, Duan K, Yang C. Effect of long-acting versus short-acting glucagon-like peptide-1 receptor agonists on improving body weight and related metabolic parameters in type 2 diabetes: A head-to-head meta-analysis. Medicine (Baltimore). 2023;102(43):e35739. doi: 10.1097/MD.0000000000035739 37904378 PMC10615555

[33] Latif W, Lambrinos KJ, Patel P. Compare and contrast the glucagon-like peptide-1 receptor agonists (GLP1RAs). StatPearls. Treasure Island (FL): StatPearls Publishing. 2025. 34283517

[34] Pasricha PJ, Parkman HP. Gastroparesis: definitions and diagnosis. Gastroenterol Clin North Am. 2015;44(1):1–7. doi: 10.1016/j.gtc.2014.11.001 25667018

[35] Kashyap P, Farrugia G. Diabetic gastroparesis: what we have learned and had to unlearn in the past 5 years. Gut. 2010;59(12):1716–26. doi: 10.1136/gut.2009.199703 20871131 PMC2996823

[36] Egboh S-M, Duncanson K, Potter M, Keely S, Talley NJ. Functional dyspepsia and gastroparesis: are they distinct disorders, a spectrum of diseases or one disease?. eGastroenterology. 2025;3(1):e100119. doi: 10.1136/egastro-2024-100119 39944931 PMC11770444

[37] Bravata D, Liu H, Colosimo MM, Bullock AC, Commons E, Pimentel M. Digital disease management programme reduces chronic gastrointestinal symptoms among racially and socially vulnerable populations. BMJ Open Gastroenterol. 2024;11(1):e001463. doi: 10.1136/bmjgast-2024-001463 39209334 PMC11367377

[38] O’Grady G, Varghese C, Andrews CN, Gharibans AA. Differentiating gastroparesis from functional dyspepsia is no longer sufficient. Gut. 2026;75(4):842–4. doi: 10.1136/gutjnl-2025-336908 40973477

[39] Aneke-Nash C, Hung KS, Wall-Wieler E, Zheng F, Sharaiha RZ. Comparing the risk of gastroparesis following different modalities for treating obesity: semaglutide versus bupropion-naltrexone versus sleeve gastrectomy - a retrospective cohort study. BMJ Open Gastroenterol. 2025;12(1):e001704. doi: 10.1136/bmjgast-2024-001704 40175094 PMC11966946

