# Supplementary material for: Gastroparesis induced by glucagon-like peptide-1 receptor agonists: A systematic review of clinical features, diagnosis, management, and outcomes
Source: PLoS One. 2026 Aug 13;21(8):e0354497. doi: 10.1371/journal.pone.0354497 (PMC13472420; doi:10.1371/journal.pone.0354497)
Supplement: Peer review history

# Author comment: Author response to Decision Letter 0

**DOI:** 10.1371/journal.pone.0354497.r001

**Transfer Alert**

This paper was transferred from another journal. As a result, its full editorial history (including decision letters, peer reviews and author responses) may not be present.

9 Mar 2026

# Peer review: Decision Letter 0

**DOI:** 10.1371/journal.pone.0354497.r002

**Authors:** Lanlan Chen

12 Jun 2026

Dear Dr. Olubodun,

Thank you for submitting your manuscript to PLOS ONE. After careful consideration, we feel that it has merit but does not fully meet PLOS ONE’s publication criteria as it currently stands. Therefore, we invite you to submit a revised version of the manuscript that addresses the points raised during the review process.

Please submit your revised manuscript by Jul 27 2026 11:59PM. If you will need more time than this to complete your revisions, please reply to this message or contact the journal office at plosone@plos.org. When you're ready to submit your revision, log on to https://www.editorialmanager.com/pone/ and select the 'Submissions Needing Revision' folder to locate your manuscript file.

- A letter that responds to each point raised by the academic editor and reviewer(s). You should upload this letter as a separate file labeled 'Response to Reviewers'.
- A marked-up copy of your manuscript that highlights changes made to the original version. You should upload this as a separate file labeled 'Revised Manuscript with Track Changes'.
- An unmarked version of your revised paper without tracked changes. You should upload this as a separate file labeled 'Manuscript'.

If you would like to make changes to your financial disclosure, please include your updated statement in your cover letter. Guidelines for resubmitting your figure files are available below the reviewer comments at the end of this letter.

If applicable, we recommend that you deposit your laboratory protocols in protocols.io to enhance the reproducibility of your results. Protocols.io assigns your protocol its own identifier (DOI) so that it can be cited independently in the future. For instructions see: https://journals.plos.org/plosone/s/submission-guidelines#loc-laboratory-protocols. Additionally, PLOS ONE offers an option for publishing peer-reviewed Lab Protocol articles, which describe protocols hosted on protocols.io. Read more information on sharing protocols at https://plos.org/protocols?utm_medium=editorial-email&utm_source=authorletters&utm_campaign=protocols.

As the corresponding author, your ORCID iD is verified in the submission system and will appear in the published article. PLOS supports the use of ORCID, and we encourage all coauthors to register for an ORCID iD and use it as well. Please encourage your coauthors to verify their ORCID iD within the submission system before final acceptance, as unverified ORCID iDs will not appear in the published article. *Only*  the individual author can complete the verification step; PLOS staff *cannot*  verify ORCID iDs on behalf of authors.

We look forward to receiving your revised manuscript.

Kind regards,

Lanlan Chen

Academic Editor

PLOS One

Journal Requirements:

When submitting your revision, we need you to address these additional requirements.

1.Please ensure that your manuscript meets PLOS ONE's style requirements, including those for file naming. The PLOS ONE style templates can be found at

https://journals.plos.org/plosone/s/file?id=wjVg/PLOSOne_formatting_sample_main_body.pdf and

https://journals.plos.org/plosone/s/file?id=ba62/PLOSOne_formatting_sample_title_authors_affiliations.pdf

2. Please ensure that you have uploaded your PRISMA flowchart as Figure 1, or else explain why this is not possible. Blank flowcharts can be found here: http://www.prisma-statement.org/.

3. Please provide a complete Data Availability Statement in the submission form, ensuring you include all necessary access information or a reason for why you are unable to make your data freely accessible. If your research concerns only data provided within your submission, please write "All data are in the manuscript and/or supporting information files" as your Data Availability Statement.

4. We note that there is identifying data in the Supporting Information file “Additional file 3_Summary Table of included studies” Due to the inclusion of these potentially identifying data, we have removed this file from your file inventory. Prior to sharing human research participant data, authors should consult with an ethics committee to ensure data are shared in accordance with participant consent and all applicable local laws.

Data sharing should never compromise participant privacy. It is therefore not appropriate to publicly share personally identifiable data on human research participants. The following are examples of data that should not be shared:

-Name, initials, physical address

-Ages more specific than whole numbers

-Internet protocol (IP) address

-Specific dates (birth dates, death dates, examination dates, etc.)

-Contact information such as phone number or email address

-Location data

-ID numbers that seem specific (long numbers, include initials, titled “Hospital ID”) rather than random (small numbers in numerical order)

Data that are not directly identifying may also be inappropriate to share, as in combination they can become identifying. For example, data collected from a small group of participants, vulnerable populations, or private groups should not be shared if they involve indirect identifiers (such as sex, ethnicity, location, etc.) that may risk the identification of study participants.

Additional guidance on preparing raw data for publication can be found in our Data Policy (https://journals.plos.org/plosone/s/data-availability#loc-human-research-participant-data-and-other-sensitive-data) and in the following article: http://www.bmj.com/content/340/bmj.c181.long.

Please remove or anonymize all personal information (<specific identifying information in file to be removed>), ensure that the data shared are in accordance with participant consent, and re-upload a fully anonymized data set. Please note that spreadsheet columns with personal information must be removed and not hidden as all hidden columns will appear in the published file.

5. If the reviewer comments include a recommendation to cite specific previously published works, please review and evaluate these publications to determine whether they are relevant and should be cited. There is no requirement to cite these works unless the editor has indicated otherwise.

Additional Editor Comments:

This study is of interest to the general readers as GLP1-RA is getting more and more important and widely prescribed. However, this manuscirpt should revise to faciliate reading and intepretation:

1. Please remove additional file 1 to the main text of methods.

2. The additional file 2 should be figure 1

3. The additional file 3 and 4 should be table 1 and 2 separately.

4. The discussion is not sufficient, please refer to the related publications (PMID: 40175094; PMID: 39209334)

[Note: HTML markup is below. Please do not edit.]

Reviewer's Responses to Questions

**Comments to the Author**

1. Is the manuscript technically sound, and do the data support the conclusions?

Reviewer #1: Yes

**********

2. Has the statistical analysis been performed appropriately and rigorously?

Reviewer #1: Yes

**********

3. Have the authors made all data underlying the findings in their manuscript fully available?

The PLOS Data policy

Reviewer #1: Yes

**********

4. Is the manuscript presented in an intelligible fashion and written in standard English?

Reviewer #1: Yes

**********

Reviewer #1: Given the rapidly expanding use of GLP-1 receptor agonists for diabetes and weight management. This systematic review summarizes reported cases of GLP-1RA-associated gastroparesis, focusing on clinical presentation, diagnostic evaluation, management, and outcomes. Overall, the review highlights the importance of recognizing persistent or severe gastrointestinal symptoms as a potential drug-related complication. However, several issues should be addressed.

The manuscript would benefit from a clearer distinction between GLP-1RA-induced gastroparesis and GLP-1RA-exacerbated pre-existing diabetic gastroparesis, particularly because several included cases had diabetes or prior symptoms suggestive of delayed gastric emptying.

Since six of the included reports were conference abstracts, the authors should discuss more explicitly how incomplete reporting may affect the reliability of patient characteristics, diagnostic details, management strategies, and outcomes.

The statement that radiological investigations and endoscopy are sufficient to diagnose GLP-1RA-induced gastroparesis may be too strong. It would be more appropriate to state that these findings can support a suspected diagnosis after mechanical obstruction has been excluded.

The manuscript states that symptom onset ranged from hours to several months after GLP-1RA initiation; however, the temporal pattern is not clearly summarized. Please consider categorizing the included cases by time to onset to better characterize the clinical presentation.

The Introduction or Discussion would benefit from a brief consideration of the clinical overlap between gastroparesis and functional dyspepsia, as symptoms such as nausea, vomiting, early satiety, bloating, and postprandial discomfort are not specific to gastroparesis (eg, PMID: 39944931 and PMID: 40973477).

The authors should consider adding a brief practical algorithm or summary pathway for clinicians, including symptom recognition, exclusion of obstruction, consideration of gastric emptying testing, GLP-1RA discontinuation, supportive treatment, and follow-up.

**********

what does this mean?). If published, this will include your full peer review and any attached files.

If you choose “no”, your identity will remain anonymous but your review may still be made public.

**Do you want your identity to be public for this peer review?**  For information about this choice, including consent withdrawal, please see our Privacy Policy

Reviewer #1: No

**********

[NOTE: If reviewer comments were submitted as an attachment file, they will be attached to this email and accessible via the submission site. Please log into your account, locate the manuscript record, and check for the action link "View Attachments". If this link does not appear, there are no attachment files.]

To ensure your figures meet our technical requirements, please review our figure guidelines: https://journals.plos.org/plosone/s/figures

You may also use PLOS’s free figure tool, NAAS, to help you prepare publication quality figures: https://journals.plos.org/plosone/s/figures#loc-tools-for-figure-preparation.

NAAS will assess whether your figures meet our technical requirements by comparing each figure against our figure specifications.

# Author comment: Author response to Decision Letter 1

**DOI:** 10.1371/journal.pone.0354497.r003

2 Jul 2026

POINT BY POINT RESPONSE

Journal Requirements:

When submitting your revision, we need you to address these additional requirements.

1.Please ensure that your manuscript meets PLOS ONE's style requirements, including those for file naming. The PLOS ONE style templates can be found at

https://journals.plos.org/plosone/s/file?id=wjVg/PLOSOne_formatting_sample_main_body.pdf and

https://journals.plos.org/plosone/s/file?id=ba62/PLOSOne_formatting_sample_title_authors_affiliations.pdf

Response: We have renamed all files correctly

2. Please ensure that you have uploaded your PRISMA flowchart as Figure 1, or else explain why this is not possible. Blank flowcharts can be found here: http://www.prisma-statement.org/.

Response: We have now uploaded PRISMA flowchart as Figure 1

3. Please provide a complete Data Availability Statement in the submission form, ensuring you include all necessary access information or a reason for why you are unable to make your data freely accessible. If your research concerns only data provided within your submission, please write "All data are in the manuscript and/or supporting information files" as your Data Availability Statement.

Response: We have written - All data are in the manuscript and/or supporting information files

4. We note that there is identifying data in the Supporting Information file “Additional file 3_Summary Table of included studies” Due to the inclusion of these potentially identifying data, we have removed this file from your file inventory. Prior to sharing human research participant data, authors should consult with an ethics committee to ensure data are shared in accordance with participant consent and all applicable local laws.

Data sharing should never compromise participant privacy. It is therefore not appropriate to publicly share personally identifiable data on human research participants. The following are examples of data that should not be shared:

-Name, initials, physical address

-Ages more specific than whole numbers

-Internet protocol (IP) address

-Specific dates (birth dates, death dates, examination dates, etc.)

-Contact information such as phone number or email address

-Location data

-ID numbers that seem specific (long numbers, include initials, titled “Hospital ID”) rather than random (small numbers in numerical order)

Data that are not directly identifying may also be inappropriate to share, as in combination they can become identifying. For example, data collected from a small group of participants, vulnerable populations, or private groups should not be shared if they involve indirect identifiers (such as sex, ethnicity, location, etc.) that may risk the identification of study participants.

Additional guidance on preparing raw data for publication can be found in our Data Policy (https://journals.plos.org/plosone/s/data-availability#loc-human-research-participant-data-and-other-sensitive-data) and in the following article: http://www.bmj.com/content/340/bmj.c181.long.

Please remove or anonymize all personal information (<specific identifying information in file to be removed>), ensure that the data shared are in accordance with participant consent, and re-upload a fully anonymized data set. Please note that spreadsheet columns with personal information must be removed and not hidden as all hidden columns will appear in the published file.

Response: Thank you for bringing this to our attention. We have carefully reviewed and revised Additional File 3 (now Table 1) to further protect participant confidentiality. Specifically, we have anonymized the participant-level data by replacing exact ages with age ranges, categorizing glycaemic control (rather than reporting exact blood glucose and HbA1c values), and removing specific laboratory results while retaining clinically relevant summaries. These revisions preserve the scientific content of the supplementary table while minimizing the risk of participant identification.

5. If the reviewer comments include a recommendation to cite specific previously published works, please review and evaluate these publications to determine whether they are relevant and should be cited. There is no requirement to cite these works unless the editor has indicated otherwise.

Response: We have noted this

ADDITIONAL EDITOR COMMENTS

This study is of interest to the general readers as GLP1-RA is getting more and more important and widely prescribed. However, this manuscirpt should revise to faciliate reading and intepretation:

1. Please remove additional file 1 to the main text of methods.

Response: Thank you. This has been done

2. The additional file 2 should be figure 1

Response: Thank you. This has been done

3. The additional file 3 and 4 should be table 1 and 2 separately.

Response: Thank you. This has been done

4. The discussion is not sufficient, please refer to the related publications (PMID: 40175094; PMID: 39209334)

Response: Thank you. We have improved on the discussion.

REVIEWER'S RESPONSES TO QUESTIONS

Comments to the Author

1. Is the manuscript technically sound, and do the data support the conclusions?

The manuscript must describe a technically sound piece of scientific research with data that supports the conclusions. Experiments must have been conducted rigorously, with appropriate controls, replication, and sample sizes. The conclusions must be drawn appropriately based on the data presented.

Reviewer #1: Yes

________________________________________

2. Has the statistical analysis been performed appropriately and rigorously?

Reviewer #1: Yes

________________________________________

3. Have the authors made all data underlying the findings in their manuscript fully available?

The PLOS Data policy requires authors to make all data underlying the findings described in their manuscript fully available without restriction, with rare exception (please refer to the Data Availability Statement in the manuscript PDF file). The data should be provided as part of the manuscript or its supporting information, or deposited to a public repository. For example, in addition to summary statistics, the data points behind means, medians and variance measures should be available. If there are restrictions on publicly sharing data—e.g. participant privacy or use of data from a third party—those must be specified.

Reviewer #1: Yes

________________________________________

4. Is the manuscript presented in an intelligible fashion and written in standard English?

PLOS ONE does not copyedit accepted manuscripts, so the language in submitted articles must be clear, correct, and unambiguous. Any typographical or grammatical errors should be corrected at revision, so please note any specific errors here.

Reviewer #1: Yes

________________________________________

5. Review Comments to the Author

Please use the space provided to explain your answers to the questions above. You may also include additional comments for the author, including concerns about dual publication, research ethics, or publication ethics. (Please upload your review as an attachment if it exceeds 20,000 characters)

Reviewer #1: Given the rapidly expanding use of GLP-1 receptor agonists for diabetes and weight management. This systematic review summarizes reported cases of GLP-1RA-associated gastroparesis, focusing on clinical presentation, diagnostic evaluation, management, and outcomes. Overall, the review highlights the importance of recognizing persistent or severe gastrointestinal symptoms as a potential drug-related complication. However, several issues should be addressed.

1. The manuscript would benefit from a clearer distinction between GLP-1RA-induced gastroparesis and GLP-1RA-exacerbated pre-existing diabetic gastroparesis, particularly because several included cases had diabetes or prior symptoms suggestive of delayed gastric emptying.

Response: Thank you for this important observation. We agree that distinguishing de novo GLP-1RA-induced gastroparesis from exacerbation of pre-existing diabetic gastroparesis is clinically relevant. We have revised the Discussion to explicitly differentiate these entities by discussing cases that were more consistent with de novo drug-induced gastroparesis and those that more likely represented exacerbation or unmasking of pre-existing diabetic gastroparesis. Specifically, we note that several included cases had documented gastroparesis, previous gastroparesis episodes, or symptoms suggestive of delayed gastric emptying before GLP-1RA initiation, and are therefore more appropriately interpreted as exacerbations of underlying disease rather than de novo drug-induced gastroparesis.

2. Since six of the included reports were conference abstracts, the authors should discuss more explicitly how incomplete reporting may affect the reliability of patient characteristics, diagnostic details, management strategies, and outcomes.

Response: Thank you for this valuable suggestion. We have revised the Limitations section to explicitly discuss the implications of including conference abstracts. Specifically, we now note that conference abstracts frequently provide limited information on patient characteristics, diagnostic investigations, management strategies, follow-up, and clinical outcomes. We also explain that incomplete reporting may have limited our ability to compare cases, distinguish de novo GLP-1RA-induced gastroparesis from exacerbation of pre-existing gastroparesis, and comprehensively evaluate management approaches and outcomes. These additions to the limitations section, provide a clearer interpretation of how the inclusion of conference abstracts may have affected the reliability and completeness of the evidence synthesized in this review.

The statement that radiological investigations and endoscopy are sufficient to diagnose GLP-1RA-induced gastroparesis may be too strong. It would be more appropriate to state that these findings can support a suspected diagnosis after mechanical obstruction has been excluded.

Response: Thank you for this important observation. We agree that our original wording overstated the diagnostic role of radiological investigations and endoscopy. We have revised the Discussion to clarify that these investigations were primarily used to demonstrate gastric distension or retained gastric contents and to exclude mechanical obstruction. We now state that, when interpreted alongside the temporal relationship between symptom onset and GLP-1RA exposure, these findings supported a suspected diagnosis of GLP-1RA-associated gastroparesis rather than being sufficient to establish the diagnosis. We have also clarified that gastric emptying scintigraphy (GES) was performed mainly in patients with chronic or longstanding symptoms to confirm delayed gastric emptying and, in some cases, to document reversibility following withdrawal of the GLP-1RA. This revision more accurately reflects the findings of the included studies and avoids overstating the diagnostic role of radiological investigations and endoscopy.

The manuscript states that symptom onset ranged from hours to several months after GLP-1RA initiation; however, the temporal pattern is not clearly summarized. Please consider categorizing the included cases by time to onset to better characterize the clinical presentation.

Response: Thank you for this helpful suggestion. We have revised both the Results and Discussion sections to provide a clearer summary of the temporal pattern of symptom onset following GLP-1RA exposure. In the Results, we categorized the cases according to the interval between GLP-1RA exposure and symptom onset, distinguishing those that developed within one week from those that developed between one and three months after treatment initiation or dose escalation, while also describing cases in which the timing could not be precisely classified. In the Discussion, we expanded the interpretation of these findings by highlighting the variability in symptom onset and emphasizing that gastroparesis may occur not only shortly after treatment initiation but also following dose escalation, inappropriate re-initiation, unsupervised use, or switching between GLP-1RA agents. These revisions better characterize the temporal pattern of presentation and its clinical implications.

The Introduction or Discussion would benefit from a brief consideration of the clinical overlap between gastroparesis and functional dyspepsia, as symptoms such as nausea, vomiting, early satiety, bloating, and postprandial discomfort are not specific to gastroparesis (eg, PMID: 39944931 and PMID: 40973477).

Response: Thank you for this helpful suggestion. We agree that the symptoms of gastroparesis overlap substantially with those of functional dyspepsia and are therefore not specific to gastroparesis. We have revised the Clinical Presentation subsection of the Discussion to acknowledge this overlap. Specifically, we now note that symptoms such as nausea, vomiting, early satiety, postprandial fullness, epigastric pain, and bloating may also occur in functional dyspepsia, and emphasize that these symptoms should be interpreted within the overall clinical context, including their temporal relationship with GLP-1RA exposure, symptom severity and persistence, and the presence of features suggestive of delayed gastric emptying. We have also incorporated the recommended references to support this discussion.

The authors should consider adding a brief practical algorithm or summary pathway for clinicians, including symptom recognition, exclusion of obstruction, consideration of gastric emptying testing, GLP-1RA discontinuation, supportive treatment, and follow-up.

Response: Thank you for this valuable suggestion. We have addressed this comment by developing and incorporating a practical clinical algorithm (Figure 2) into the manuscript. The algorithm provides a concise, evidence-based summary of the recognition and management of suspected GLP-1RA-associated gastroparesis, including symptom recognition, assessment of the temporal relationship to GLP-1RA therapy, exclusion of alternative causes (including mechanical obstruction), diagnostic evaluation, GLP-1RA discontinuation, supportive treatment, and follow-up. We have also added a new subsection entitled "Practical clinical approach" in the Discussion to introduce the figure and highlight its intended use as a pragmatic clinical aid derived from the evidence synthesized in this systematic review, rather than as a formal clinical guideline.

Attachment Submitted filename: Response to reviewers.docx

# Peer review: Decision Letter 1

**DOI:** 10.1371/journal.pone.0354497.r004

**Authors:** Lanlan Chen

8 Jul 2026

Gastroparesis induced by glucagon-like peptide-1 receptor agonists: a systematic review of clinical features, diagnosis, management, and outcomes

PONE-D-26-11854R1

Dear Dr. Olubodun,

We’re pleased to inform you that your manuscript has been judged scientifically suitable for publication and will be formally accepted for publication once it meets all outstanding technical requirements.

Within one week, you’ll receive an e-mail detailing the required amendments. When these have been addressed, you’ll receive a formal acceptance letter and your manuscript will be scheduled for publication.

An invoice will be generated when your article is formally accepted. Please note, if your institution has a publishing partnership with PLOS and your article meets the relevant criteria, all or part of your publication costs will be covered. Please make sure your user information is up-to-date by logging into Editorial Manager at Editorial Manager® and clicking the ‘Update My Information' link at the top of the page. For questions related to billing, please contact billing support.

If your institution or institutions have a press office, please notify them about your upcoming paper to help maximize its impact. If they’ll be preparing press materials, please inform our press team as soon as possible -- no later than 48 hours after receiving the formal acceptance. Your manuscript will remain under strict press embargo until 2 pm Eastern Time on the date of publication. For more information, please contact onepress@plos.org.

Kind regards,

Lanlan Chen

Academic Editor

PLOS One

Additional Editor Comments (optional):

Reviewers' comments:

Reviewer's Responses to Questions

**Comments to the Author**

Reviewer #1: All comments have been addressed

**********

2. Is the manuscript technically sound, and do the data support the conclusions?

Reviewer #1: Yes

**********

3. Has the statistical analysis been performed appropriately and rigorously?

Reviewer #1: Yes

**********

4. Have the authors made all data underlying the findings in their manuscript fully available?

The PLOS Data policy

Reviewer #1: Yes

**********

5. Is the manuscript presented in an intelligible fashion and written in standard English?

Reviewer #1: Yes

**********

Reviewer #1: After carefully reading and evaluating this manuscript, I have no additional revision suggestions or concerns to raise for the authors.

**********

what does this mean?). If published, this will include your full peer review and any attached files.

If you choose “no”, your identity will remain anonymous but your review may still be made public.

**Do you want your identity to be public for this peer review?**  For information about this choice, including consent withdrawal, please see our Privacy Policy

Reviewer #1: No

**********

# Editor report: Acceptance letter

**DOI:** 10.1371/journal.pone.0354497.r005

**Authors:** Lanlan Chen

PONE-D-26-11854R1

PLOS One

Dear Dr. Olubodun,

I'm pleased to inform you that your manuscript has been deemed suitable for publication in PLOS One. Congratulations! Your manuscript is now being handed over to our production team.

At this stage, our production department will prepare your paper for publication. This includes ensuring the following:

* All references, tables, and figures are properly cited

* All relevant supporting information is included in the manuscript submission,

* There are no issues that prevent the paper from being properly typeset

You will receive further instructions from the production team, including instructions on how to review your proof when it is ready. Please keep in mind that we are working through a large volume of accepted articles, so please give us a few days to review your paper and let you know the next and final steps.

Lastly, if your institution or institutions have a press office, please let them know about your upcoming paper now to help maximize its impact. If they'll be preparing press materials, please inform our press team within the next 48 hours. Your manuscript will remain under strict press embargo until 2 pm Eastern Time on the date of publication. For more information, please contact onepress@plos.org.

You will receive an invoice from PLOS for your publication fee after your manuscript has reached the completed accept phase. If you receive an email requesting payment before acceptance or for any other service, this may be a phishing scheme. Learn how to identify phishing emails and protect your accounts at https://explore.plos.org/phishing.

If we can help with anything else, please email us at customercare@plos.org.

Thank you for submitting your work to PLOS ONE and supporting open access.

Kind regards,

PLOS ONE Editorial Office Staff

on behalf of

M.D Lanlan Chen

Academic Editor

PLOS One
